# Erythropoietin alleviates hepatic insulin resistance via PPARγ-dependent AKT activation

**DOI:** 10.1038/srep17878

**Published:** 2015-12-08

**Authors:** Zhijuan Ge, Pengzi Zhang, Ting Hong, Sunyinyan Tang, Ran Meng, Yan Bi, Dalong Zhu

**Affiliations:** 1 Department of Endocrinology, DrumTower hospital affiliated to Nanjing University Medical School, No321 Zhongshan Road, Nanjing, 210008, China; 2 Department of Endocrinology, Drum Tower Clinical Medical College of Nanjing Medical University, Nanjing, 210008, China

## Abstract

Erythropoietin (EPO) has beneficial effects on glucose metabolism and insulin resistance. However, the mechanism underlying these effects has not yet been elucidated. This study aimed to investigate how EPO affects hepatic glucose metabolism. Here, we report that EPO administration promoted phosphatidylinositol 3-kinase (PI3K)/AKT pathway activation in palmitic acid (PA)-treated HepG2 cells and in the liver of high-fat diet (HFD)-fed mice, whereas adenovirus-mediated silencing of the erythropoietin receptor (EPOR) blocked EPO-induced AKT signalling in HepG2 cells. Importantly, a peroxisome proliferator-activated receptor γ (PPARγ) antagonist and PPARγ small interfering RNA (siRNA) abrogated the EPO-induced increase in p-AKT in HepG2 cells. Lentiviral vector-mediated hepatic PPARγ silencing in HFD-fed C57BL/6 mice impaired EPO-mediated increases in glucose tolerance, insulin sensitivity and hepatic AKT activation. Furthermore, EPO activated the AMP-activated protein kinase (AMPK)/sirtuin 1 (SIRT1) signalling pathway, and AMPKα and SIRT1 knockdown each attenuated the EPO-induced PPARγ expression and deacetylation and PPARγ-dependent AKT activation in HepG2 cells. In summary, these findings suggest that PPARγ is involved in EPO/EPOR-induced AKT activation, and targeting the PPARγ/AKT pathway via EPO may have therapeutic implications for hepatic insulin resistance and type 2 diabetes.

Erythropoietin (EPO), a glycoprotein hormone produced in the foetal liver and adult kidney, stimulates erythropoiesis by binding to its specific cell surface receptor (EPOR) on erythroid progenitor cells^1^,^2^. EPOR is expressed in nonerythroid tissues and cells, including the brain^3^, kidney^4^, liver^5^, pancreatic islet^6^, and endothelial cells^7^, suggesting that EPO has other biological activities beyond erythropoiesis. EPO also has beneficial effects on glucose metabolism and insulin resistance. EPO administration ameliorated glucose metabolism in haemodialysis patients^8^,^9^ and reduced blood glucose levels in healthy mice, as well as in murine diabetes and obesity models^5^,^10^,^11^,^12^. In contrast, EPOR-null mice developed insulin resistance and glucose intolerance in non-erythroid tissues or adipose tissue^13^,^14^, and endogenous EPO levels are reduced in patients with type 2 diabetes^15^. EPO/EPOR signalling may modulate glucose metabolism via activation of the phosphatidylinositol 3-kinase (PI3K)/AKT pathway in adipocytes *in vitro* and *in vivo*^14^,^16^,^17^. However, the effect of EPO on insulin signalling and glucose metabolism in hepatocytes, as well as the underlying mechanism, remains poorly understood.

Peroxisome proliferator-activated receptor γ (PPARγ) is a ligand-activated nuclear receptor and transcription factor that is expressed in adipose tissue, liver and muscle^18^,^19^. This factor is involved in lipid metabolism via its regulation of genes, such as fatty acid binding protein 2 (aP2) and lipoprotein lipase (LPL)^20^. PPARγ is also crucial for controlling the expression of genes involved in glucose homeostasis, including the first member of family with sequence similarity 3 gene family (FAM3A), phosphoenolpyruvate carboxykinase (PEPCK) and PI3K^20^,^21^,^22^. PPARγ agonist-mediated PI3K/AKT pathway stimulation reportedly mediates its insulin sensitizing effect in adipocytes and hepatocytes^20^,^23^,^24^,^25^. Conversely, mice with a liver-specific knockout of PPARγ displayed hyperlipidaemia and elevated plasma glucose^19^,^26^. These studies indicate that PPARγ plays a vital role in the regulation of hepatic glucose metabolism.

In this study, we demonstrated that EPO/EPOR signalling promoted AKT activation in HepG2 cells and in livers of high-fat diet (HFD)-fed mice. Moreover, both small interfering RNA (siRNA)-mediated PPARγ knockdown in palmitic acid (PA)-treated HepG2 cells and lentivirus-mediated knockdown of hepatic PPARγ in HFD-fed C57BL/6 mice attenuated EPO-induced hepatic AKT activation. Furthermore, EPO also promoted activation of the AMP-activated protein kinase (AMPK)/sirtuin 1 (SIRT1) pathway, which mediates PPARγ activity to stimulate AKT signalling in HepG2 cells in response to EPO. Our findings are the first to demonstrate that EPO improves hepatic insulin resistance via PPARγ-dependent AKT activation.

## Methods

### Antibodies

Antibodies against PI3K-p85, AKT, p-AKT (Ser473), IRS2, phosphorylated glycogen synthase kinase-3β (p-GSK3β) (Ser9), phosphorylated forkhead box O1 (p-FOXO1) (Ser256), AMPKα, p-AMPKα (Thr172) and liver kinase B1 (LKB1) were all purchased from Cell Signaling Technology (Danvers, MA, USA). Anti-SIRT1 and anti-EPOR antibodies were obtained from Santa Cruz Biotechnology (Dallas, Texas, USA). Antibodies against PPARγ, GSK3β, FOXO1 and β-actin were obtained from Bioworld Technology (St. Louis Park, MN, USA). The anti-p-IRS2 (Ser731) antibody was from AnaSpec (Fremont, CA, USA).

### Cell culture

HepG2 cells were purchased from American Type Culture Collection (Rockville, MD, USA), cultured in DMEM supplemented with 10% FBS at 37 °C in a humidified atmosphere of 95% air and 5% CO_2_. After reaching 70% confluence, cells were incubated in serum-free medium for 8 h before treatment. Cells were then treated with 0.3 mmol/l PA, 10 U/ml EPO, or 12.5 μmol/l rosiglitazone for 24 h, as indicated. For insulin stimulation, cells were incubated for another 3 h with 100 nmol/l insulin before harvest unless otherwise noted. Where indicated, cells were pretreated with 10 μmol/l PPARγ antagonist (GW9662, Sigma-Aldrich, Saint Louis, MO, USA), 20 μmol/l AMPK inhibitor (Compound C, Calbiochem, Darmstadt, Germany) or 10 μg/ml Ca^2+^/calmodulin-dependent kinase kinase (CaMKK) inhibitor (STO-609, Calbiochem) for 2 h prior to EPO treatment. The appropriate vehicle served as the controls. Cell lysates were collected for western blot analysis.

### RNA interference (RNAi)

To knockdown EPOR in HepG2 cells, cells were infected with an adenoviral vector expressing an EPOR short hairpin RNA (shRNA) sequence (Genechem, Shanghai, China) or an AdCMV-GFP control vector (Genechem) for 48 h prior to treatment with PA or EPO. To silence SIRT1 or PPARγ gene expression, HepG2 cells were transfected with siRNA against SIRT1 or PPARγ (GenePharma, Shanghai, China) using Lipofectamine 2000 (Invitrogen, Grand Island, NY, USA) according to the manufacturer’s guidelines. Luciferase siRNA (FAM) was used as a control. The siRNA oligonucleotides are as follows: SIRT1, 5′-GCGGGAAUCCAAAGGAUAATT-3′; PPARγ, 5′-ACUCCACAUUACGAAGACATT-3′; AMPKα, 5′-CCACUCUCCUGAUGCAUAUTT-3′; LKB1, 5′-CCUGCUGAAAGGGAUGCUUTT-3′; and negative control FAM, 5′-UUCUCCGAACGUGUCACGUTT-3′. If indicated, 0.3 mmol/l PA and 10 U/ml EPO were added 36 h post-transfection.

### Animals

Four-week-old male C57BL/6 mice were purchased from the Comparative Medicine Centre of Yangzhou University (Yangzhou, China) and maintained on a 12-h light-dark cycle with free access to food and drinking water. The mice were fed a HFD (60 kcal% fat, 20 kcal% carbohydrates, and 20 kcal% protein, Guangzhou Animal Experiment Centre, Guangzhou, China) or a normal chow diet (NC, 10 kcal% fat, 70 kcal% carbohydrates, 20 kcal% protein) for 12 weeks. To knockdown hepatic PPARγ in HFD-fed C57BL/6 mice, recombinant lentiviruses encoding PPARγ shRNA (Lv-PPARγ-shRNA) or scrambled negative control shRNA (Lv-NC) produced by Genechem were applied. The PPARγ shRNA sequence was 5′-CCGGGCCCTGGCAAAGCATTTGTATCTCGAGATACAAATGCTTTGCCAGGGCTTTTTG-3′.HFD-fed mice were then randomised to receive an injection of 1 × 10^7^ plaque-forming units (PFU) recombinant lentivirus (Lv-PPARγ-shRNA or Lv-NC) or no injection via the tail vein one week prior to intraperitoneal treatment with 2000 IU/kg recombinant human EPO (Sunshine Pharmaceutical, Shenyang, China) every other day. HFD control mice were injected with PBS as a vehicle control. After two weeks of EPO treatment, the mice were deprived of food for 8 h and killed for collection of the fasting blood and tissue. All of the animal procedures were performed according to the National Institutes of Health guidelines and approved by the animal care committee of Drum Tower Hospital, which is affiliated with Nanjing University Medical School, Nanjing, China.

### Glucose tolerance tests (GTTs) and insulin tolerance tests (ITTs)

Both GTTs and ITTs were performed after 2 weeks of EPO administration. For the GTT, the mice were fasted for 10 h and challenged with an intraperitoneal glucose load (1.5 g/kg wt). For the ITT, mice received an intraperitoneal insulin injection (0.8 U/kg wt, Novolin R, Novo Nordisk Inc., Copenhagen, Denmark) after 4 h of fasting. Blood glucose levels were measured via the tail vein at 0, 30, 60, and 120 min after the glucose or insulin load with a glucometer (Accu-Chek Active, Roche Diagnostic, Germany).

### Fasting plasma insulin measurement

Fasting insulin levels were measured using an ELISA kit according to the manufacturer’s instructions (Millipore, Billerica, MA, USA).

### Western blotting

Cells were lysed in RIPA buffer that had been supplemented with Protease Inhibitor Cocktail (Roche, Basel, Switzerland), and cell debris was removed by centrifugation at 14000 × g at 4 °C for 15 min. Protein concentrations were quantitated using a BCA protein assay. Sample proteins were separated by 8% SDS-PAGE and transferred to polyvinylidenedifluoride (PVDF) membranes (Millipore), which were blocked with 5% nonfat milk in Tris-buffered saline containing 0.1% Tween 20 (TBST) and probed by incubation with primary antibodies at 4 °C overnight. After three washes with TBST, the membranes were incubated with HRP-conjugated secondary antibodies for 2 h, followed by detected using enhanced chemiluminescence (Millipore). Band intensities were quantified using Quantity One (Bio-Rad, Hercules, CA, USA).

### Analysis of glycogen content

HepG2 cells were pretreated with the PPARγ antagonist GW9662 (Sigma-Aldrich) for 2 h prior to PA and EPO treatment. After a 24-h incubation, cells were treated with or without 100 nmol/l insulin for a final 3 h, and the amount of intracellular glycogen was then determined using a glycogen assay kit (BioVision, Milpitas, CA, USA).

### Quantitative real-time PCR

Total RNA was extracted from HepG2 cells using TRIzol reagent (Invitrogen). RNA was reverse transcribed to cDNA using a PrimeScript RT reagent Kit (Takara Bio, Otsu, Japan). Real-time PCR was performed on an ABI PRISM 7500 Sequence Detection System (Applied Biosystems, Grand Island, NY, USA) with SYBR Premix Ex Taq (Takara Bio). Primer sequences are listed in supplemental Table 1. The expression level of each test gene was normalised to β-actin as an internal control.

### NAD^+^/NADH ratio assay

NAD^+^ and NADH levels were determined from whole-cell extracts of HepG2 cells using a NAD^+^/NADH Quantification Colorimetric Kit (BioVision) according to the manufacturer’s instructions.

### Statistical analysis

The data were expressed as the mean ± SE. Differences among groups were analysed by one-way ANOVA with the least significant difference (LSD) or Dunnett T3 post hoc comparison analysis as appropriate. Values of P < 0.05 were considered statistically significant.

## Results

### EPO/EPOR activates AKT signalling in hepatocytes

**Figure 1:**
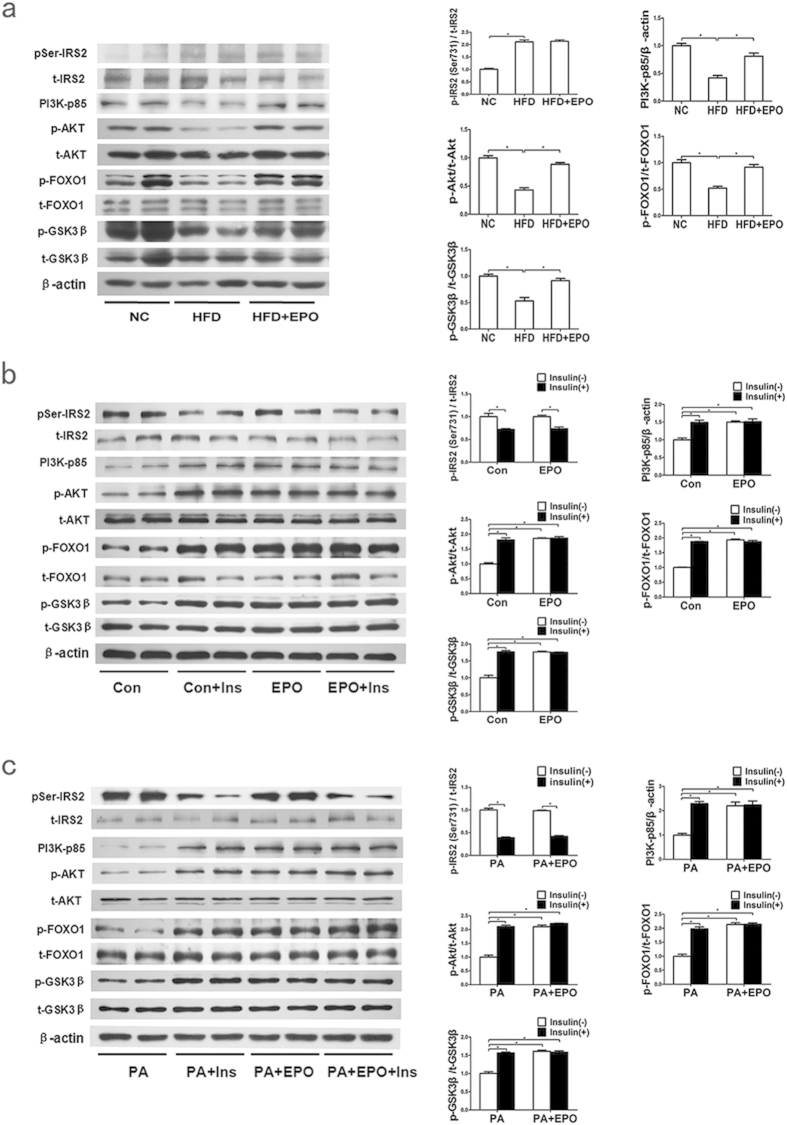
EPO activates AKT signalling in hepatocytes. (**a**) The hepatic protein expression of pIRS2/IRS2, PI3K-p85, p-AKT/AKT, p-FOXO1/FOXO1 and p-GSK3β/GSKβ was determined by western blotting in HFD-fed mice with or without EPO treatment. NC: normal chow diet; HFD: high-fat diet. Data are expressed as the mean ± SE (n = 5); *P < 0.05 (**b,c**) Protein levels were determined by western blotting in normal (**b**) and PA-induced (**c**) HepG2 cells that had been treated with EPO (10 U/ml) either in the absence (white bars) or presence of insulin (100 nmol/L, black bars). The graphs show the mean ± SE (n = 3); *P < 0.05.

The PI3K/AKT pathway is a crucial regulator of hepatic glucose metabolism, and impaired phosphorylation of AKT and its substrates GSK3β and FOXO1 increases glycogen synthesis and decreases gluconeogenesis, respectively^27^,^28^. As shown in Fig. 1a, in HFD-fed C57BL/6 mice, hepatic protein levels of PI3K-p85, p-AKT, p-FOXO1 and p-GSK3β increased significantly after EPO treatment. Similarly, in both control- and PA-treated HepG2 cells, EPO administration increased the PI3K-p85, p-AKT, p-FOXO1 and p-GSK3β expression to a similar extent as induced by insulin; these levels were not further elevated by treatment with EPO and insulin together (Fig. 1b,c). Nonetheless, EPO did not alter the p-IRS2 (Ser731) and IRS2 (Fig. 1a–c) protein levels. These results suggest that EPO has a beneficial effect on the AKT signalling pathway in hepatocytes.

**Figure 2:**
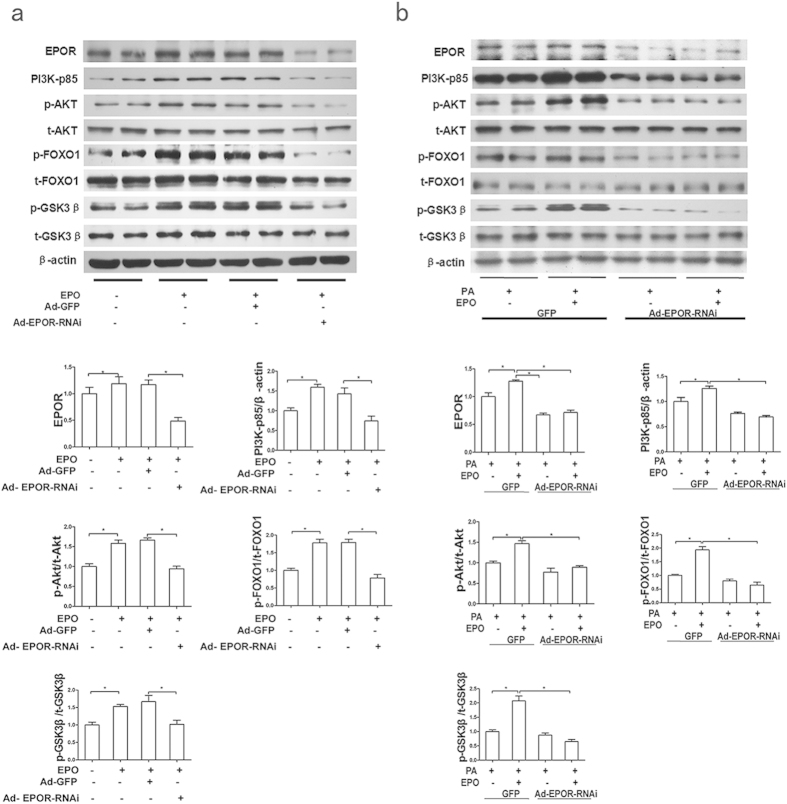
EPOR is required for EPO-mediated activation of the AKT signalling pathway. (**a,b**) The effects of EPOR RNAi on EPOR, PI3K-p85, p-AKT/AKT, p-FOXO1/FOXO1 and p-GSK3β/GSKβ protein expression were determined by western blotting after EPO treatment in both normal (**a**) and PA-treated (**b**) HepG2 cells. The graphs show the mean ± SE (n = 3); *P < 0.05.

To investigate whether EPO-induced AKT activation was mediated by EPOR, we utilized RNAi to knockdown EPOR expression in HepG2 cells. As shown in Fig. 2a,b, adenovirus-mediated EPOR silencing significantly blunted the EPO-induced increases in PI3K-p85 and p-AKT protein levels compared with that in control transfected HepG2 cells in either normal or PA-treated conditions. Meanwhile, EPOR knockdown greatly diminished the EPO-induced change in p-FOXO1 and p-GSK3β protein levels. These results suggest that EPOR expression permits a direct response to EPO in HepG2 cells.

### PPARγ mediates EPO-induced AKT activation to improve hepatic insulin resistance

**Figure 3:**
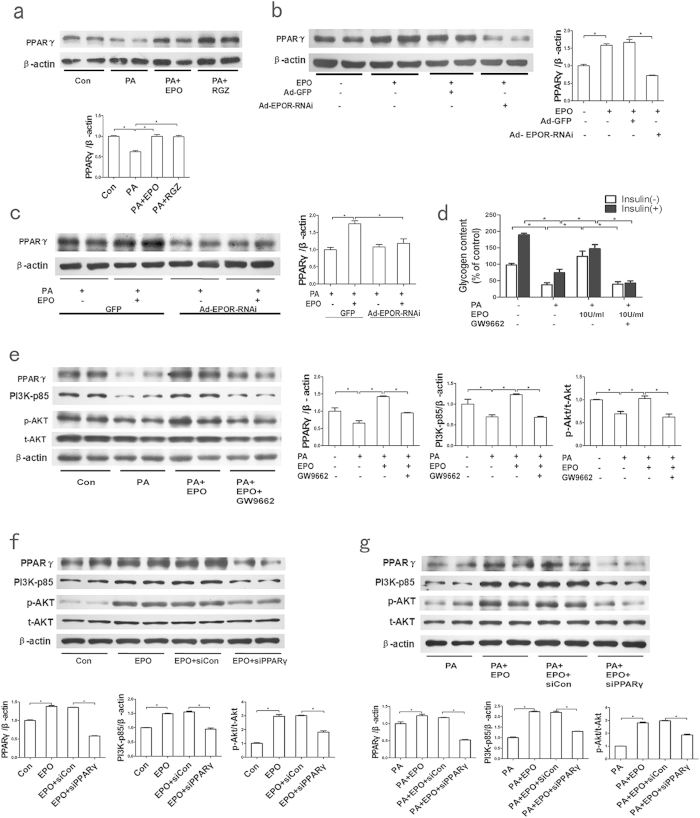
PPARγ mediates EPO-induced AKT activation. (**a**) The PPARγ protein levels were measured by western blotting in EPO-treated PA-induced HepG2 cells. (**b,c)** The effects of EPOR RNAi on PPARγ protein levels were determined by western blotting after EPO treatment in both normal (**b**) and PA-treated (**c**) HepG2 cells. (**d**) Cellular glycogen levels were measured in PA-induced HepG2 cells that had been pretreated with GW9662 prior to EPO administration with or without insulin treatment. (**e**) The PPARγ, PI3K-p85 and p-AKT/AKT protein expression levels were determined by western blotting after EPO treatment in PA-induced HepG2 cells with or without GW9662 treatment. (**f,g**) The effects of siRNA-PPARγ on PPARγ, PI3K-p85 and p-AKT/AKT protein levels upon EPO treatment were determined in both normal (**f**) and PA-treated (**g**) cells. The graphs show the mean ± SE (n = 3); *P < 0.05. RGZ: rosiglitazone.

PPARγ activation reportedly increases p-AKT levels, possibly by inducing PI3K expression *in vivo* in rodents and *in vitro* in cell lines^23^,^24^,^29^, but it is unknown whether PPARγ activation is required for EPO-induced AKT activation in hepatocytes. First, the effect of EPO treatment on PPARγ expression was assessed. The results demonstrated that EPO significantly increased the PPARγ protein expression in PA-treated HepG2 cells, an effect that was similar to that of the PPARγ agonist rosiglitazone (Fig. 3a). Adenovirus-mediated EPOR silencing significantly blunted the EPO-induced increases in PPARγ protein levels in normal and PA-treated HepG2 cells (Fig. 3b,c).

**Figure 4:**
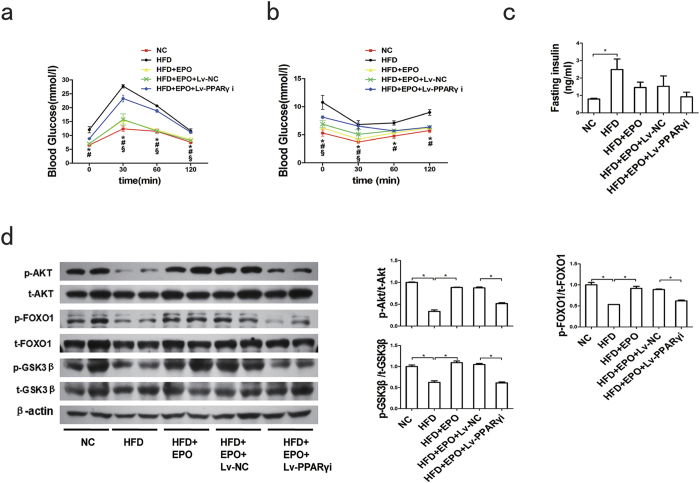
Loss of hepatic PPARγ decreases the EPO-induced maintenance of glucose homeostasis and activation of hepatic insulin signalling in HFD-fed mice (**a,b**) A glucose tolerance test (GTT) (**a**) and insulin tolerance test (ITT) (**b**) were performed after EPO treatment in HFD-fed mice lacking liver PPARγ and in controls. Values are the mean ± SE (n = 6). (**c**) Fasting plasma insulin levels were determined after EPO treatment in HFD-fed mice lacking hepatic PPARγ and in controls. (**d**) The hepatic p-AKT/AKT, p-FOXO1/FOXO1 and p-GSK3β/GSKβ protein expression was determined by western blotting after EPO treatment in HFD-fed mice lacking hepatic PPARγ and in controls. Data are expressed as the mean ± SE (n = 4–5); *P < 0.05. For (**a,b**), “*”means HFD versus NC, P < 0.05; “#” means HFD + EPO versus HFD, P < 0.05; “§” means HFD + EPO + Lv-PPARγi versus HFD + EPO + Lv-NC, P < 0.05. NC: normal chow diet; HFD: high-fat diet; Lv-NC: mice injected with lentivirus encoding scrambled negative control shRNA via the tail vein; Lv-PPARγi: mice injected with lentivirus encoding shRNA targeting PPARγ via the tail vein.

Additionally, to determine whether PPARγ is involved in the capacity of EPO to restore PA-inhibited insulin signalling, HepG2 cells were pretreated with the PPARγ antagonist GW9662 prior to EPO administration. GW9662 abrogated the EPO-induced increases in PI3K-p85 and p-AKT protein levels and glycogen levels (Fig. 3d,e) in PA-treated cells. PPARγ siRNA was transfected into HepG2 cells to knockdown PPARγ. Compared with control siRNA transfection, transfection with siRNA-PPARγ blocked the EPO-induced PI3K-p85 and p-AKT expression in both normal and PA-treated cells (Fig. 3f,g). To further confirm the role of PPARγ in EPO-induced AKT activation *in vivo*, lentivirus vector-based RNAi was used to knockdown hepatic PPARγ in HFD-fed C57BL/6 mice without upregulation of PPARα (supplemental Fig. 1). The results demonstrated that EPO-mediated increases in glucose tolerance, insulin sensitivity and hepatic p-AKT, p-FOXO1 and p-GSK3β protein levels were sharply attenuated by hepatic PPARγ silencing in HFD-fed mice (Fig. 4a,b,d). Fasting insulin levels did not significantly change after EPO treatment in HFD-fed mice lacking hepatic PPARγ or in controls (Fig. 4c). These results indicate that PPARγ mediates EPO-induced hepatic AKT activation *in vitro* and *in vivo*.

### CaMKK/AMPK/SIRT1 is required for EPO-mediated PPARγ activation

**Figure 5:**
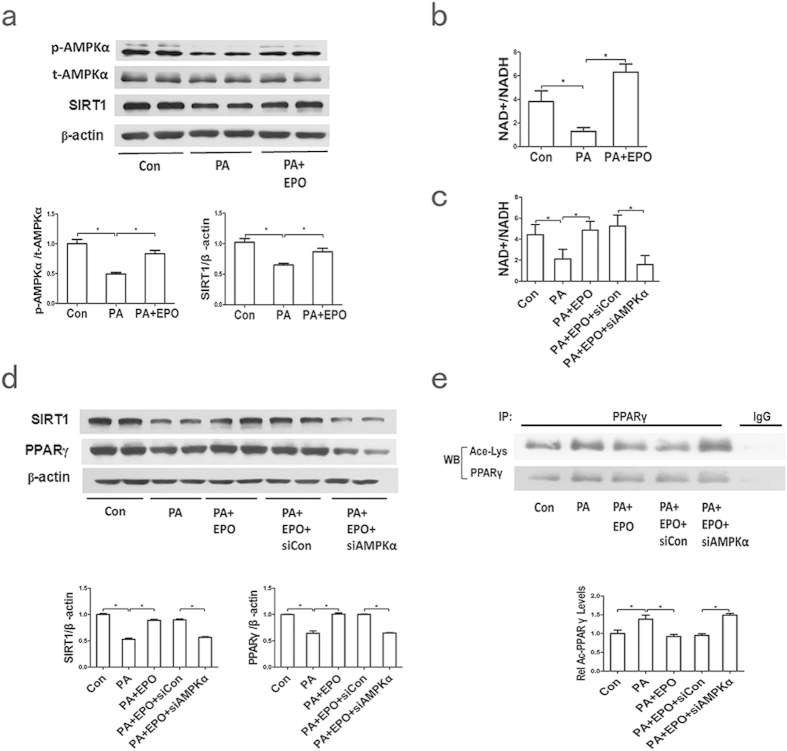
AMPK activity is involved in EPO-induced activation of SIRT1 and PPARγ. (**a**) The p-AMPKα/AMPKα and SIRT1 protein expression was determined by western blotting after EPO treatment in PA-induced HepG2 cells. (**b**) The NAD^ + ^/NADH ratio was determined after EPO treatment in PA-induced HepG2 cells. (**c,d**) The effects of siRNA-AMPKα on the NAD^ + ^/NADH ratio (**c**) and the SIRT1 and PPARγ protein levels (**d**) after EPO treatment were determined in PA-treated cells. (**e**) The effects of siRNA-AMPKα on PPARγ deacetylation levels after EPO treatment were determined in PA-treated cells. The graphs show the mean ± SE (n = 3); *P < 0.05.

**Figure 6:**
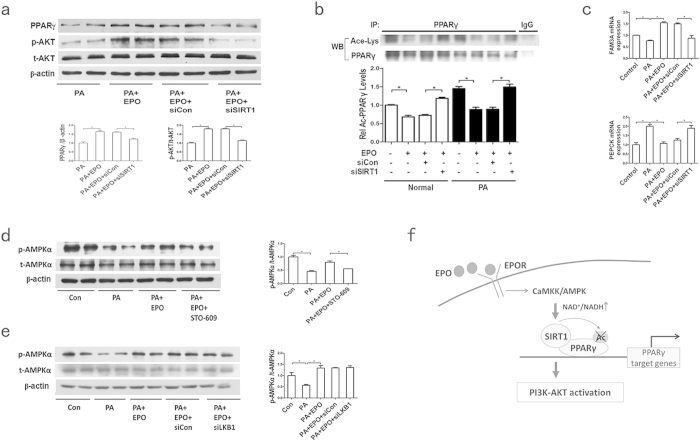
CaMKK/AMPK/SIRT1 is required for PPARγ-dependent AKT activation after EPO treatment. (**a**) The effects of siRNA-SIRT1 on the PPARγ and p-AKT/AKT protein levels after EPO treatment were determined in PA-treated cells. (**b**) The effects of siRNA-SIRT1 on PPARγ deacetylation levels after EPO treatment were determined in PA-treated cells. (**c**) The effects of SIRT1 siRNA on the mRNA levels of FAM3A and PEPCK after EPO treatment were determined by quantitative RT-PCR in PA-treated cells. (**d,e**) The p-AMPKα/AMPKα protein expression was determined by western blotting after EPO treatment in PA-induced HepG2 cells that had been treated with or without the CaMKK inhibitor STO-609 (**d**) or LKB1 siRNA (**e**). (**f**) A model based on our studies: EPO enhanced hepatic PPARγ activity via the activation of CaMKK/AMPK/SIRT1 signaling, which results in PI3K/AKT activation to improve hepatic insulin resistance. Graphs show the mean ± SE (n = 3); *P < 0.05.

SIRT1, a NAD^+^-dependent protein deacetylase, is a vital protein that plays a role in metabolic homoeostasis. SIRT1 activity can be enhanced by AMPK activation through increases in the NAD^+^/NADH ratio^30^. Previous studies demonstrated that SIRT1 upregulates PPARγ activity via deacetylation and PPARγ expression^31^,^32^,^33^, but it is unknown whether AMPK/SIRT1 is responsible for the PPARγ-dependent AKT activation upon EPO treatment. We observed that EPO treatment upregulated AMPK activity (p-AMPKα) and SIRT1 protein levels and the NAD^+^/NADH ratio in PA-treated HepG2 cells (Fig. 5a,b). Moreover, AMPK activity inhibition using the AMPK inhibitor Compound C and knockdown of AMPKα (supplemental Fig. 2a) each attenuated the EPO-induced increases in the NAD^+^/NADH ratio and the SIRT1 protein expression and PPARγ deacetylation (supplemental Fig. 3a–c, Fig. 5c–e), suggesting that AMPK activity was involved in the EPO-induced activation of SIRT1 and PPARγ. Additionally, HepG2 cells were pretreated with SIRT1 siRNA (supplemental Fig. 2b) prior to EPO administration. The results demonstrated that the EPO-induced increases in the PPARγ protein and deacetylation levels and the AKT phosphorylation were abrogated after SIRT1 silencing in PA-treated cells (Fig. 6a,b). SIRT1 silencing also influenced the EPO-induced changes in the mRNA levels of PPARγ target genes, including FAM3A and PEPCK, in HepG2 cells (Fig. 6c). These observations indicate that the AMPK/SIRT1 pathway may be involved in the EPO-mediated effects on PPARγ-dependent AKT activation.

LKB1 and CaMKK are two major AMPK kinases that activate AMPK activity^34^,^35^. Therefore, we investigated whether CaMKK or LKB1 could mediate the EPO-induced AMPK activity in HepG2 cells that had been pretreated with the CaMKK inhibitor STO-609 or LKB1 siRNA (supplemental Fig. 2c) before EPO treatment. As shown in Fig. 6d,e, EPO-mediated AMPK activation was hampered by the CaMKK inhibitor but not by LKB1 silencing, indicating that EPO may activate AMPK via CaMKK but not LKB1.

Together, these data suggest that the beneficial effect of EPO on hepatic AKT activation requires a CaMKK/AMPK/SIRT1-mediated increase in PPARγ activity.

## Discussion

Evidence has indicated that EPO attenuates obesity and diabetes in mouse models^10^,^11^,^13^ and promotes energy metabolism in adipocytes^14^,^36^ and fat oxidation in muscle^37^. Here, we report for the first time that EPO treatment enhances hepatic AKT pathway activity to improve insulin resistance via a PPARγ-dependent mechanism.

Previous studies have demonstrated that EPO/EPOR signalling activates IRS2 and PI3K/AKT in erythroid cells, but the downstream pathways in nonerythroid cells have not yet been elucidated. Recent studies demonstrated that EPO administration increased AKT but not IRS phosphorylation to improve insulin resistance in 3T3L1 adipocytes^16^,^17^ and to promote pancreatic β cell growth and survival^11^. In this study, we observed that EPO stimulated AKT activation but not IRS2 activation in PA-treated HepG2 cells and the livers of HFD-fed mice (Fig. 1a–c); this finding is consistent with our previous study reporting AKT activation by EPO in the livers of HFD-fed mice^5^. Furthermore, the EPO-induced increases in PI3K-p85 and p-AKT protein levels were blocked by EPOR knockdown in HepG2 cells (Fig. 2a,b), indicating that the PI3K/AKT pathway is involved in EPO/EPOR signalling. Furthermore, EPO stimulation increased the p-GSK3β and p-FOXO1 protein levels and cellular glycogen levels in hepatocytes (Figs 1a–c, 3d), suggesting that EPO can regulate hepatic glucose homeostasis via AKT signalling.

PPARs, which include PPARα, PPARβ/δ, and PPARγ, are transcription factors that belong to the nuclear hormone receptor superfamily, heterodimerize with retinoid X receptor and regulate genes involved in lipid/glucose homoeostasis^38^,^39^. A recent study demonstrated that EPO increases PPARα activity and expression to increase energy expenditure and induce a thermogenic gene program in white adipocytes^14^. Our results revealed that EPO/EPOR upregulates PPARγ expression to alleviate insulin resistance in hepatocytes (Fig. 3a–c). PPARγ regulates the expression of genes encoding key insulin signalling enzymes. PPARγ agonists stimulated the PI3K/AKT pathway by enhancing PI3K expression and activity and AKT phosphorylation in rodents *in vivo* and in adipocyte and hepatocyte cell lines *in vitro*^20^,^23^,^24^,^25^,^40^. PPARγ overexpression increased p-AKT levels and glycogen synthesis in insulin-resistant skeletal myocytes^41^. In this study, both the PPARγ antagonist GW9662 and PPARγ silencing using PPARγ siRNA abrogated EPO-stimulated increases in PI3K-p85 and p-AKT expression in PA-treated HepG2 cells (Fig. 3e–g). Furthermore, GW9662 also diminished EPO-induced increases in cellular glycogen levels (Fig. 3d). Importantly, the EPO-induced increases in glucose tolerance and hepatic protein levels of p-AKT, p-FOXO1 and p-GSK3β were also significantly decreased after knockdown of hepatic PPARγ in HFD-fed C57BL/6 mice *in vivo* (Fig. 4a,b,d). These observations indicated that EPO may alleviate hepatic insulin resistance via activation of the PPARγ-dependent PI3K/AKT pathway. Our results also revealed a significant reduction in the liver triglyceride content after five weeks of EPO treatment in ob/ob mice (supplemental Fig. 4). Recent studies in our laboratory further suggested that EPO administration attenuated hepatic steatosis, possibly by repressing the protein expression of fatty acid translocase (CD36), fatty acid binding protein-1 (FABP1) and sterol regulatory element binding protein-1c (SREBP1c); these proteins are involved in the uptake, intracellular transport and synthesis of fatty acids. EPO treatment also increased the protein levels of carnitine palmitoyltransferase 1 (CPT1), which is involved in fatty acid oxidation in ob/ob mice (Zhijuan Ge *et al*. unpublished data). Collectively, our data indicated that EPO treatment for two to five weeks could upregulate PPARγ to activate the PI3K/AKT pathway and thus alleviate hepatic insulin resistance without inducing hepatic lipid deposition.

Next, we investigated how EPO activates PPARγ in hepatocytes. SIRT1, a NAD^+^-dependent protein deacetylase, plays a central role in glucose and lipid homeostasis in mammals. SIRT1 modulates endocrine signalling by deacetylating lysine residues on histones and many transcriptional factors, such as PPARγ coactivator (PGC-1α), PPARγ and PPARα^31^,^32^,^42^,^43^,^44^. AMPK upregulates SIRT1 expression and activity by increasing the intracellular NAD^+^/NADH ratio^30^. Two recent studies demonstrated that EPO stimulated the AMPK/SIRT1 pathway to promote mitochondrial function and protect against oxidative stress in white adipose tissue^14^,^36^. Here, we demonstrated that EPO increased SIRT1 protein levels and activity through increases in the NAD^+^/NADH ratio in HepG2 cells, which were accompanied by an increase in AMPK phosphorylation (Fig. 5a,b). Furthermore, both inhibition and suppression of AMPKα resulted in decreases in the NAD^+^/NADH ratio, the SIRT1 expression and the PPARγ protein and deacetylation levels in response to EPO (supplemental Fig. 3a–c, Fig. 5c–e). Moreover, our data demonstrated that SIRT1 silencing abrogated EPO-induced increases in PPARγ protein and deacetylation levels and AKT phosphorylation (Fig. 6a,b). The mRNA levels of PPARγ target genes, including FAM3A and PEPCK, in response to EPO were also influenced by the SIRT1 knockdown (Fig. 6c). Thus, EPO may enhance SIRT1 activity by increasing the AMPK-dependent NAD^+^/NADH ratio, resulting in PPARγ deacetylation and modulation of its activity. A previous study indicated that SIRT1 positively regulated mTORC, which led to AKT phosphorylation in the liver^45^. Therefore, we investigated whether EPO-induced SIRT1 activation affects mTORC. We determined that EPO did not affect mTOR expression in PA-treated HepG2 cells (supplemental Fig. 5). These data suggest that EPO-induced SIRT1 activation may activate AKT via PPARγ but not via the mTOR pathway. EPO also reportedly induced Ca^2+^ influx in erythroblasts, myoblasts and neurons^36^,^46^. Our data also demonstrated that EPO-induced AMPK activation was hampered by CaMKK inhibitor treatment but not by LKB1 knockdown (Fig. 6d,e), indicating that EPO might increase Ca^2+^ influx to activate CaMKK, thereby activating AMPK. These observations support the hypothesis that PPARγ may be regulated by EPO via regulation of CaMKK/AMPK/SIRT1 pathway activity to activate AKT in hepatocytes (Fig. 6f).

In conclusion, the current study demonstrates that EPO/EPOR signalling activates the hepatic AKT pathway by increasing PPARγ expression and activity. Our study demonstrates for the first time that PPARγ is essential for EPO-induced improvements in hepatic insulin resistance, which supports the idea that EPO may contribute to a novel strategy for treating insulin resistance and type 2 diabetes.

## Additional Information

**How to cite this article**: Ge, Z. *et al*. Erythropoietin alleviates hepatic insulin resistance via PPARγ-dependent AKT activation. *Sci. Rep*. **5**, 17878; doi: 10.1038/srep17878 (2015).

## Supplementary Material

Supplementary Information
