# Supplementary material for: Erythropoietin alleviates hepatic insulin resistance via PPARγ-dependent AKT activation
Source: Sci Rep. 2015 Dec 8;5:17878. doi: 10.1038/srep17878 (PMC4672330; doi:10.1038/srep17878)
Supplement: Supplementary Information [file srep17878-s1.pdf]

Supplementary information

Erythropoietin alleviates hepatic insulin resistance via PPARγ-dependent AKT activation

Zhijuan Ge[1, +], Pengzi Zhang[1, +], Ting Hong[2, +], Sunyinyan Tang[1], Ran Meng[1], Yan Bi[1, *], Dalong Zhu[1, *]

[*] Corresponding authors: Yan Bi, biyan@nju.edu.cn; Dalong Zhu, zhudalong@nju.edu.cn.

[+] These authors contributed equally to this work

Supplemental Methods

Determination of hepatic triglyceride content. A 50 mg sample of frozen liver tissue was minced and homogenised in 1 ml tissue lysis buffer, and the triglyceride content was measured using a tissue triglyceride assay kit (Applygen Tec, Beijing, China) according to the manufacturer's instructions.

Supplemental Table 1. Gene-specific primer sequences for real-time PCR analysis

Gene	Forward primer	Reverse primer
FAM3A	GTGTCACATGGATCGTGGTC	TGCTCAATCAGCATCTTGTCC
PEPCK	CCTGCGGCTTAACTGGCAT	GTGGCAGTATTCTCAGCCTCA
β-ACTIN	CATGTACGTTGCTATCCAGGC	CTCCTTAATGTCACGCACGAT

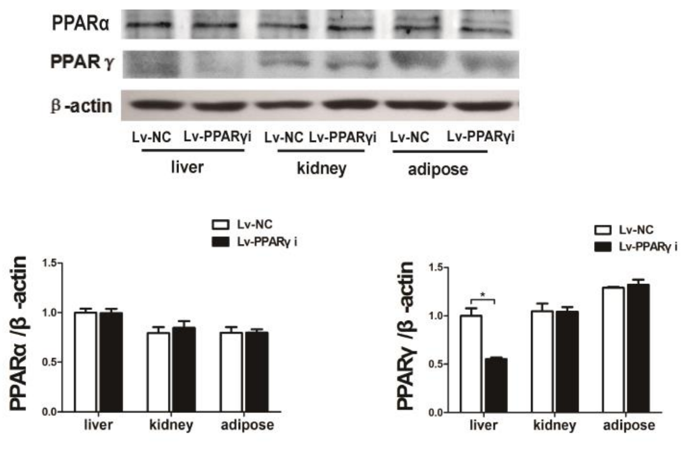

Supplemental Figure 1. Lentiviral vector-mediated knockdown of hepatic PPARγ in

HFD-fed C57BL/6 mice. PPARγ and PPARα protein levels in liver, kidney and adipose tissues were measured by western blotting in C57BL/6 mice that had been injected with a lentivirus against PPARγ via the tail vein. Data are expressed as the mean±SE (n=4); *P<0.05. Lv-NC: mice that were injected with a lentivirus encoding scrambled negative control shRNA via the tail vein; Lv-PPARγi: mice that had been injected with a lentivirus encoding shRNA targeting PPARγ via the tail vein.

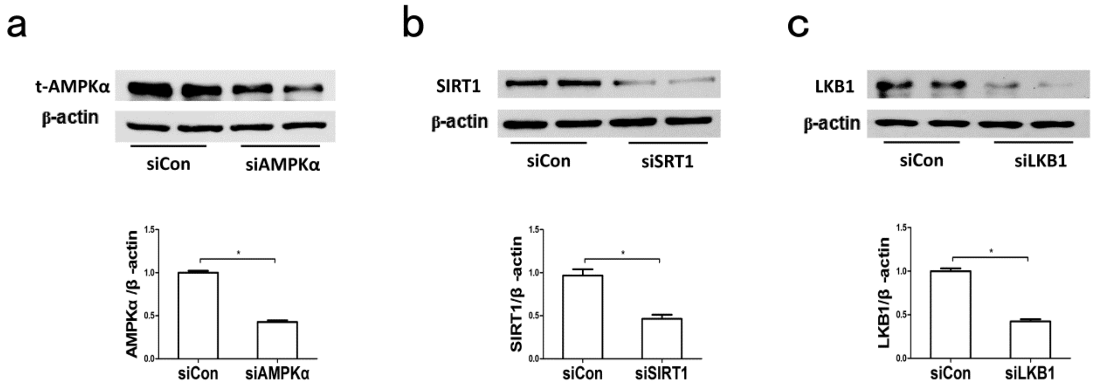

Supplemental Figure 2. Confirmation of siRNA-mediated knockdown of AMPKα (siAMPKα), SIRT1 (siSIRT1) or LKB1 (siLKB1). AMPKα **(a),** SIRT1 **(b)** or LKB1 **(c)** protein levels were determined by western blotting. The graphs show the mean±SE (n=3); *P<0.05.

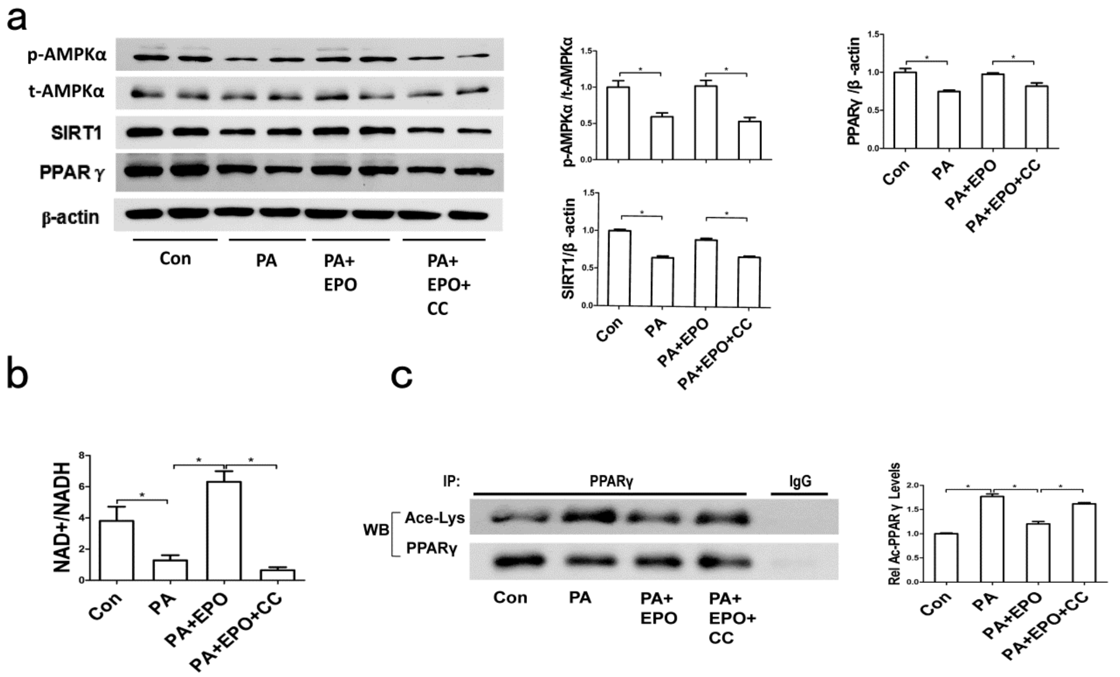

Supplemental Figure 3. EPO-induced increases in the NAD$^+$/NADH ratio, SIRT1 protein expression and PPARγ deacetylation levels are attenuated by the AMPKα inhibitor Compound C. (a) The p-AMPKα/AMPKα, SIRT1 and PPARγ protein expression was determined by western blotting after EPO treatment in PA-induced HepG2 cells that had been treated with or without Compound C. **(b)** The NAD$^+$/NADH ratio was determined after EPO treatment in PA-induced HepG2 cells that had been treated with or without Compound C. **(c)** PPARγ deacetylation levels after EPO treatment were determined in PA-treated cells that had been treated with or without Compound C. The graphs show the mean ±SE (n=3); *P<0.05. CC: Compound C.

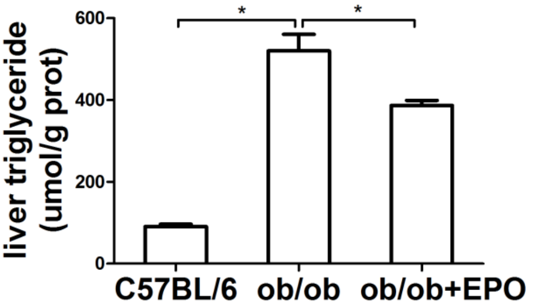

Supplemental Figure 4. EPO treatment significantly reduces the lipid deposition in the livers of ob/ob mice. The liver triglyceride content was assessed in ob/ob mouse livers after five weeks of EPO treatment or no treatment. Data are expressed as the mean±SE (n=6); *P<0.05.

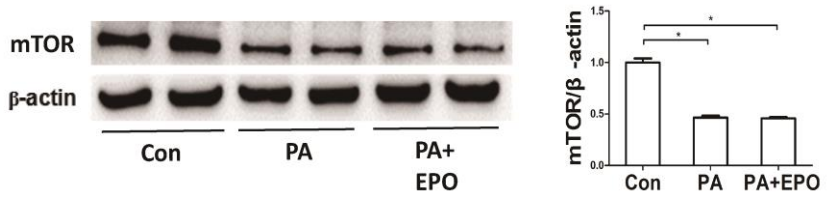

Supplemental Figure 5. EPO treatment has no effect on mTOR expression in PA-treated HepG2 cells. The mTOR protein expression was determined by western blotting after EPO treatment in PA-induced HepG2 cells. The graphs show the mean±SE (n=3); *P<0.05.
